# Chlorin e6 mediated photodynamic inactivation for multidrug resistant *Pseudomonas aeruginosa* keratitis in mice *in vivo*

**DOI:** 10.1038/srep44537

**Published:** 2017-03-15

**Authors:** Ming-Feng Wu, Mona Deichelbohrer, Thomas Tschernig, Matthias W. Laschke, Nóra Szentmáry, Dirk Hüttenberger, Hans-Jochen Foth, Berthold Seitz, Markus Bischoff

**Affiliations:** 1 Department of Ophthalmology, Saarland University Medical Center, Homburg/Saar, Germany; 2 Department of Ophthalmology, Union Hospital, Tongji Medical College, Huazhong University of Science and Technology, Wuhan, China; 3 Institute for Anatomy and Cell Biology, Saarland University, Homburg/Saar, Germany; 4 Institute for Clinical & Experimental Surgery, Saarland University, Homburg/Saar, Germany; 5 Department of Ophthalmology, Semmelweis University, Budapest, Hungary; 6 ApocarePharma GmbH, Bielefeld, Germany; 7 Department of Physics, University of Kaiserslautern, Kaiserslautern, Germany; 8 Institute for Medical Microbiology and Hygiene, Saarland University, Homburg/Saar, Germany

## Abstract

Following corneal epithelium scratches, mouse corneas were infected with the multidrug resistant (MDR) *P. aeruginosa* strain PA54. 24 hours later, 0% (for control group), 0.01%, 0.05% or 0.1% Chlorin e6 (Ce6), a second generation photosensitizer derived from chlorophyll, was combined with red light, for photodynamic inactivation (PDI). 1 hour or 2 days later, entire mouse eyes were enucleated and homogenized for counting colony forming units (CFU) of *P. aeruginosa*. For comparison, 0.1% Ce6 mediated PDI was started at 12 hours post infection, and 0.005% methylene blue mediated PDI 24 hours post infection. Clinical scores of corneal manifestation were recorded daily. Compared to the control, CFU 1 hour after PDI started 24 hours post infection in the 0.01% Ce6 and 0.05% Ce6 groups were significantly lower (more than one *log*_10_ reduction), the CFU 2 days post PDI higher in the 0.1% Ce6 group, clinical score lower in the 0.1% Ce6 group at 1 day post PDI. These findings suggest that PDI with Ce6 and red light has a transient efficacy in killing MDR-PA *in vivo*, and repetitive PDI treatments are required to fully resolve the infection. Before its clinical application, the paradoxical bacterial regrowth post PDI has to be further studied.

*Pseudomonas aeruginosa (P. aeruginosa*), a Gram-negative rod-shaped bacterium, is among the most common pathogens of bacterial keratitis^1^. *P. aeruginosa* keratitis usually causes a rapidly progressive, suppurative corneal infiltration, ulceration, and finally perforation and blindness^2^. Infections caused by *P. aeruginosa* are further complicated by the fact that multidrug resistant (MDR) variants of this species are on the rise worldwide^3^,^4^, which are difficult to treat by conventional therapy, the empirical but intensive topical treatment with commonly used ophthalmic antibiotics, such as the fluoroquinolones ciprofloxacin and ofloxacin, the aminoglycosides gentamicin and tobramycin, and the 1^st^ generation cephalosporin cefazolin, respectively^5^,^6^. Many risk factors, such as contact lens use, compromised ocular surface and preservative-free lubricant ointment use, can predispose patients to *P. aeruginosa* keratitis^7^. *P. aeruginosa* may also cause keratitis after keratoplasty, which often leads to a very poor outcome^8^. The development of alternative therapy options to fight this type of infection has become imperative.

Photodynamic inactivation (PDI) is a novel promising antimicrobial strategy against MDR bacteria^9^,^10^. It is a photochemical reaction combining light, a photosensitizer, and oxygen to generate reactive oxygen species (ROS), such as singlet oxygen (^1^O_2_) and hydroxyl radicals (HO)^11^. Unlike antibiotics, PDI causes a non-specific bactericidal effect, since ROS can damage various vital bacterial structures, mainly cell wall proteins and membranes^12^,^13^.

PDI might be especially applicable for the treatment of infectious keratitis, since the transparency and superficial location of the cornea facilitate the deposition of the photosensitizer and the irradiation process. As early as in the 1970s, some clinical studies already tried to treat viral keratitis with PDI, but these investigations were soon suspended due to severe adverse reactions, such as phototoxic epithelial keratitis and anterior uveitis^14^,^15^. In the following decades, only *in vitro* studies that explored the possibility of PDI to combat clinically important corneal pathogens were reported^16^,^17^,^18^,^19^,^20^,^21^,^22^. To date, the *in vivo* use of PDI as antimicrobial therapy is mainly restricted to the treatment of dermatological and dental infections^23^. In ophthalmology, PDI was rarely used as a treatment option for ocular infections, although another photodynamic therapy procedure, i.e. corneal crosslinking with riboflavin and ultraviolet light, is nowadays commonly applied as a treatment for corneal ectatic diseases^24^. A small number of clinical case reports indeed suggest that corneal crosslinking might be used as a therapy option for bacterial keratitis^25^,^26^,^27^,^28^, while other case reports indicated an increase in microbial infections as a complication of this therapy form when treating corneal ectatic diseases^29^,^30^,^31^,^32^,^33^,^34^,^35^,^36^,^37^,^38^. To date, only little experimental evidence is provided, whether PDI is a useful option to fight bacterial keratitis. In 2011, Shih *et al*.^39^ demonstrated the bactericidal efficiency of a combination of the antibiotic amikacin and PDI with the photosensitizer methylene blue (MB) and red light (λ = 650 nm) in a rabbit model of *Mycobacterium fortuitum* keratitis. However, they did not assess the impact of PDI as a single treatment. In the same year, El-Laithy *et al*.^40^ treated *Staphylococcus aureus* keratitis in rabbits with the photosensitizer hematoporphyrin and red light (λ = 630 nm). However, they failed to detect a statistically significant impact of this therapy regimen.

In the present study, we evaluated the efficiency of PDI with the photosensitizer Ce6 and red light (λ = 670 nm) as a single therapy option for the treatment of MDR *P. aeruginosa* keratitis in mice. Previous *in vitro* studies demonstrated that this photosensitizer/light combination can kill up to 100% of the challenged *P. aeruginosa* cells^41^,^42^,^43^,^44^. However, whether such a remarkable bactericidal rate can be achieved *in vivo* is questionable. Under *in vitro* conditions, bacteria can be fully mixed with the photosensitizer, and the generated ROS will predominantly damage the bacterial cell components. The *in vivo* environment is much more complicated. Corneal epithelium and stroma may hinder the diffusion of the photosensitizer towards the bacteria within the infected tissue. ROS can diffuse for only short distance, e.g. less than 50 nm, once they are generated from the activated photosensitizer^45^. Thus, a substantial proportion of ROS produced by PDI is likely to react with host components, including cellular structures and molecules, and may cause unexpected cascade reactions^46^.

To assess the effect of Ce6 mediated PDI as a single therapy for the treatment of MDR-PA keratitis in mice *in vivo*, three regimens of PDI were tested (Fig. 1). In all regimen, visible red light (λ = 670 nm) was applied, thereby avoiding the potential genotoxicity induced by ultraviolet light used in corneal crosslinking^47^.

In **regimen I**, at 24 h post MDR-PA infection, corneal epithelia were removed from infected mouse eyes using a hockey epithelium removal knife to improve the diffusion of the photosensitizer into the stroma^48^, before PDI mediated by 0%, 0.01%, 0.05%, and 0.1% (w/v) Ce6 and red light was applied. Mice were euthanized either 1 h post PDI or 48 h post PDI, the infected eyes enucleated and subsequently homogenized with a manual disperser for CFU determinations as outlined in Material and Methods.

In **regimen II**, PDI mediated by 0% and 0.1% Ce6 was carried out 12 h post infection on deepithelialized corneas, and mice were euthanized 60 h post PDI for CFU counting. Inflammatory cells, such as neutrophils, which are recruited to the cornea due to the *P. aeruginosa* infection, are essential for controlling the bacterial population. However, an excessive influx and persistence of these inflammatory cells may also lead to corneal destruction^2^. PDI may interfere with the corneal response to *P. aeruginosa* unintentionally by killing these inflammatory cells. Of note, Tanaka *et al*.^49^ reported that a MB concentration of around 100 μM, when activated with a red light dose of 20 J/cm^2^, spared neutrophils but killed most of the bacteria under *in vitro* conditions.

Therefore, in **regimen III**, we used MB as a reference to investigate whether it causes a similar effect as Ce6. PDI mediated by 0.005% MB was carried out at 24 h post infection. Mice were euthanized 48 h post PDI for CFU counting.

## Results

### Regimen I (Ce6 mediated PDI at 24 h post infection)

In the first regimen, PA54 infected eyes were treated 24 h post infection by PDI with different concentrations of Ce6 and red light, and bacterial loads were subsequently determined in homogenates of whole eyes enucleated 1 h and 48 h post PDI treatment, respectively, in order to determine the short term and long term effects of PDI on the bacterial loads in the infected eyes (Fig. 2). The CFU values found in the eyes of PA54 infected mice 1 h after PDI are shown in Fig. 2A. Upon PDI the bacterial loads found in mice treated with the 0.01% Ce6 gel (Log_10_CFU = 5.19 ± 0.75, *P* = 0.028) and the 0.05% Ce6 gel (Log_10_CFU = 5.13 ± 0.61, *P* = 0.015) decreased significantly when compared to the CFU rates found in the eyes of control mice (Log_10_CFU = 6.21 ± 0.91) treated with the gel-base (0% Ce6) only. CFU of 0.1% Ce6 gel treated mice (Log_10_CFU = 5.41 ± 0.63) were likewise lower than the CFU of sham treated controls (i.e. 0% Ce6), however, this difference was not statistically significant (*P* = 0.058).

A different picture emerged when the bacterial loads of PA54 infected mice were determined 48 h post PDI (Fig. 2B). Here, the bacterial loads in the eyes of mice treated with the 0.1% Ce6 gel (Log_10_CFU = 7.38 ± 0.22) increased significantly when compared with the CFU rates found in the eyes of the sham treated control group (Log_10_CFU = 6.95 ± 0.25, *P* = 0.011). Likewise, CFU rates found in the eyes of the 0.05% Ce6 gel treated group (Log_10_CFU = 7.19 ± 0.13), and the 0.01% Ce6 gel treated group (Log_10_CFU = 7.11 ± 0.27) exceeded the CFU values observed in the eyes of the sham-treated control group, however, this was not statistically significant (*P* = 0.068 and 0.333, respectively).

The course of infection in the eyes of mice challenged with PA54 was additionally monitored by stereo-microscopic determinations and graded as outlined in Materials and Methods. The clinical scores from PA54 infected eyes determined 24 h, 48 h, and 72 h post infection, respectively, are shown in Fig. 3, and representative stereo-microscopic images of PA54 infected eyes over time are given in Fig. 4. Here, significant increases in infection severity were observed for the sham treated group and the 0.01% Ce6 group, when the development of infection symptoms over time within a treatment group were compared. The clinical scores of 0.01% Ce6 gel treated mice increased significantly throughout the observation period (*P* = 0.026 for 24 h vs. 48 h, and *P* = 0.026 for 24 h vs. 72 h). Similarly, the clinical scores of sham treated mice increased significantly from 24 to 72 h post infection (*P* = 0.009). However, such increases in severity of infection over time were neither seen in 0.05% Ce6 nor in 0.1% Ce6 treated mice.

When the clinical scores for a given time point were compared between groups, in most cases no clear differences in infection symptoms were observed (*P* ≥ 0.209). The only exception was seen in the 0.1% Ce6 group, in which clinical scores at 48 h post infection (clinical score = 2.3 ± 0.5) were significantly lower than those seen with the sham treated control group (clinical score = 3.5 ± 0.5, *P* = 0.015). At 72 h post infection (i.e. 48 h post PDI), clinical scores of the 0.1% Ce6 group also displayed lower values (clinical score = 2.8 ± 0.8) than those seen in the control group (clinical score = 3.8 ± 0.4), although this was not statistically significant (*P* = 0.060).

### Regimen II (chlorin e6 mediated PDI at hour 12 post infection)

In the second regimen, PA54 infected eyes were treated 12 h post infection by PDI with 0.1% Ce6 and red light, and bacterial loads were subsequently determined in homogenates of whole eyes enucleated 60 h post PDI treatment (Fig. 5). This regimen was chosen in order to determine whether an earlier PDI treatment time point might prevent an overgrowth of surviving bacteria that was seen at 72 h post infection in regimen I. Interestingly, when PDI with 0.1% Ce6 and red light was applied earlier in infection (i.e. at 12 h post infection), the CFU values observed in the eyes of mice that were treated with 0.1% Ce6 (Log_10_CFU = 7.20 ± 0.16) were no longer different from those seen in mice of the sham treated control group (Log_10_CFU = 6.65 ± 0.67, *P* = 0.101). Similarly, the clinical scores observed 72 h post infection in mice treated by PDI with 0.1% Ce6 and red light (clinical score = 3.7 ± 0.5) were not significantly different from those seen in the sham treated control group (clinical score = 4.0 ± 0.0, *P* = 0.394) (Fig. 6). Moreover, clinical scores increased significantly from hour 12 to day 2 (*P* = 0.002), and from hour 12 to day 3 (*P* = 0.002) in both groups, when the development of infection symptoms over time within a treatment group were compared. Representative corneal manifestations of mice are shown in Fig. 7.

### Regimen III (methylene blue mediated PDI at 24 h post infection)

The photosensitizer MB is known to kill *P. aeruginosa in vitro* upon activation by red light at concentrations of about 100 μM (equivalent to 0.003%), while immune cells are largely spared by this treatment^49^. To test whether the increased outgrowth of PA54 seen in the eyes of mice treated with the 0.1% Ce6 gel at hour 72 post infection (Fig. 2B) might have been caused by an increased impairment of immune cells, we next tested the impact of PDI by MB and red light (λ = 670 nm) at 24 h post infection to eradicate this pathogen *in vivo* in our infective keratitis model. To this end, only the bacterial loads 48 h post PDI were determined (Fig. 8). PDI by 0.005% MB and red light applied 24 h post infection yielded in CFU values (Log_10_CFU = 7.25 ± 0.28) that were largely comparable to those seen in the control group (Log_10_CFU = 6.98 ± 0.15, *P* = 0.060). Likewise, the clinical scores observed 48 h and 72 h post infection in mice treated by PDI with 0.005% MB and red light (clinical scores = 3.8 ± 0.4 and 3.7 ± 0.5 for 48 and 72 h post infection) were not significantly different from those seen in the sham treated control group (clinical scores = 3.7 ± 0.5 and 4.0 ± 0.0 for 48 and 72 h post infection, *P* > 0.394) (Fig. 9). However, when the development of infection symptoms over time within a treatment group were compared, clinical scores of the sham treated control group increased significantly from hour 24 to day 3 (*P* = 0.002), while clinical scores of the 0.005% MB group increased significantly from hour 24 to day 2 (*P* = 0.041). Representative corneal manifestations of mice are shown in Fig. 10.

## Discussion

Our current study assessed the effectivity of corneal PDI by the photosensitizer Ce6 and red light (λ = 670 nm) against MDR-PA keratitis in a murine infection model. We observed that a single PDI treatment with this photosensitizer/light combination significantly decreased the bacterial load in infected eyes by more than 90% at one h post PDI when Ce6 concentrations of 0.01% and 0.05% were utilized, indicating that this PDI procedure exhibits a bactericidal effect in the mouse cornea *in vivo* as it does *in vitro*^41^,^42^,^43^,^44^,^50^.

An interesting finding is that the highest killing efficiency of PDI by Ce6/red light was not achieved by the highest concentration of Ce6, i.e. 0.1%, but rather by lower Ce6 concentrations. Such a nonlinear correlation between concentration and photodynamic inactivation effect has been observed for many photosensitizers^49^, and is in line with our previous *in vitro* findings, indicating a dose-dependent bactericidal effect of PDI by Ce6 and red light against clinical *P. aeruginosa* isolates^44^. An explanation for this finding might be that an excess of the photosensitizer decreases the transmission of light required for efficient singlet oxygen generation^49^. Alternatively, higher Ce6 concentrations might induce the expression of export systems in *P. aeruginosa* resulting in a decreased concentration of the photosensitizer at/in the bacterial cell. However, the molecular mechanisms responsible for decreased susceptibility of *P. aeruginosa* cells against PDI in presence of increased concentrations of Ce6 still need to be clarified.

The observations of equal to higher CFU values in the eyes of Ce6 treated mice at 72 h post infection (i.e. 48 h post PDI treatment) demonstrated on the other hand that a single PDI treatment was insufficient in resolving the infection *in vivo*. Especially the significant bacterial overgrowth induced by the 0.1% Ce6 PDI treatment at day 3 of regimen I is a matter of concern, although this bacterial overgrowth was not seen when Ce6 concentrations ≤0.05% were utilized. This finding is supported by previous clinical case reports, indicating an increased risk of microbial infections as a complication that may be induced by corneal crosslinking^29^,^30^,^31^,^32^,^33^,^34^,^35^,^36^,^37^,^38^.

A solution to the possible bacterial overgrowth is crucial for the long-term safety of corneal PDI. A safe corneal PDI can be applied repeatedly on the infected cornea until all bacteria are eradicated, which certainly maximizes the potency. To explore a safer PDI protocol, we carried out the experiments of regimen II and regimen III. Since *P. aeruginosa* overgrowth, the main problem, was on day 3, we only measured the CFU on day 3 in regimen II and regimen III.

In regimen II, we focused on 0.1% Ce6, as only this photosensitizer concentration induced a significant bacterial overgrowth in regimen I. When animals were treated once by PDI with 0.1% Ce6 at 12 h post infection, the clinical scores on day 3 did no longer differ from those seen in the eyes of the sham treated control group, suggesting that the risk of bacterial overgrowth might be decreased when PDI can be applied early in infection.

An inconsistency between the milder corneal symptom and higher bacterial load was found in the group treated by 0.1% Ce6 mediated PDI on day 3 of regimen I. A possible explanation might be that a higher proportion of inflammatory cells (mainly neutrophils) recruited to the focus of corneal infection were killed by this PDI regimen, leading to a reduced inflammatory response at the later stage of infection, however, at the cost of bacterial overgrowth.

To test this hypothesis, we used the photosensitizer MB in combination with red light for PDI, which was reported to spare neutrophils at concentrations of about 100 μM, and compared its effectivity to kill *P. aeruginosa* in the murine corneal scratch model with that of Ce6 mediated PDI^49^. Taking into account the insufficient diffusion of this photosensitizer into the corneal stroma^51^, the molar concentration of 0.005% MB used in regimen III was 156 μM, and thus slightly higher than the 100 μM MB recommended in a previous *in vitro* study^49^, but just equivalent to 0.009% Ce6. Moreover, for the same amount of substance, the ROS generated from irradiated MB is just about 80% of that of Ce6^37^. Clinical scores of the group treated by MB mediated PDI were not different from those of the control group, suggesting that the corneal infiltration was not markedly affected by this PDI treatment. Yet in regimen III, the group treated by MB mediated PDI showed a considerable tendency of bacterial overgrowth on day 3, compared with the control group (*P* = 0.060), while the group treated by 0.01% Ce6 mediated PDI did not show such a bacterial overgrowth on day 3 (*P* = 0.333). This finding is supported by the previous study that shows higher efficiency of Ce6 in comparison with MB in PDI of Gram-positive bacteria *in vitro*^52^. Since MB is not better than Ce6 in preventing bacterial overgrowth either, further study is required to compare more different photosensitizers.

Considering the potential of PDI as treatment option against infectious keratitis induced by MDR bacteria, additional experiments are warranted to better uncover the mechanism(s) that promote the bacterial regrowth after PDI treatment. The overall susceptibility of a pathogen to PDI may also have a profound effect on the bacterial regrowth after PDI. PDI of Gram-negative bacteria is more challenging than of Gram-positive bacteria, because Gram-negative bacteria are surrounded by a less permeable envelop consisting of an inner cytoplasmic membrane and an outer membrane, while Gram-positive bacteria are surrounded by a single cytoplasmic membrane and a porous cell wall^53^. The overall outer membrane permeability of *P. aeruginosa* is even lower than that of most other Gram-negative bacteria, for example, 12 to 100-fold lower than that of *Escherichia coli*^54^. Thus, PDI might be particularly suited as therapy option against keratitis caused by MDR Gram-positive bacteria.

After all, Ce6, and additionally MB, are just two of the most basic photosensitizers applied for PDI. A large number of novel photosensitizers have been developed recently. In some *in vitro* studies, it has been suggested that polycationic conjugates of poly-L-lysine and Ce6 have higher efficiency for Gram-negative bacteria than free Ce6^42^,^55^. Some photosensitizers even allow selective targeting and killing bacteria in the presence of mammal cells^56^,^57^,^58^. The development of the latter group of photosensitizers can not only improve the antibacterial killing potency, but also very likely to affect the safety of PDI. We are looking forward to further studies that address the *in vivo* efficiency of these novel substances against bacterial corneal infections.

In comparison with PDI, many other studies looked at different experimental approaches for treating *P. aeruginosa* keratitis. An animal study has compared the efficacy of antibiotics to treat *P. aeruginosa* keratitis^59^, and found that moxifloxacin, levofloxacin and ciprofloxacin were equally effective against the *P. aeruginosa* isolate tested. However, when keratitis is caused by a fluoroquinolone-resistant *P. aeruginosa* isolate, which are on the rise in many regions of the world, this class of antibiotics won’t be any longer a valid therapy option. Some other novel tools, such as bacteriophages and antimicrobial peptides (AMPs), are now under development, and each tool has its own risks and advantages. A recent study indicated for example the efficacy of bacteriophage KPP12 for the treatment of *P. aeruginosa* keratitis *in vivo*^60^. However, this study demonstrated that the bacteriophage failed to lyse about one third of the clinical *P. aeruginosa* strains tested, indicating that this therapy is likely to fail in a large number of cases. The AMP esculentin-1a(1–21)NH2, a frog skin-derived peptide, was also recently reported to significantly reduce the severity of infection in a murine *P. aeruginosa* keratitis model^61^, a three *log*_*10*_ reduction was achieved when the AMP was applied three times per day for four days post infection. However, the authors noted that the disease was still progressive even under this intensive regimen.

In summary, our current study has proven that Ce6 mediated PDI is able to cause about one *log*_10_ reduction of the MDR-PA cell pool in a murine keratitis model. However, the high numbers of bacterial cells seen at later stages post PDI suggest that consecutive rounds of PDI are required to reduce the amount of bacterial cells in infected eyes in a way that the host immune system can control the infection. The differences in bacterial growth observed after PDI with increasing photosensitizer concentration indicate furthermore that care needs to be taken to identify the optimal dose of photosensitizer to effectively decrease the bacterial burden without causing too many side effects that may interfere with bacterial clearance.

## Materials and Methods

### Materials

Agar plate of Trypticase^TM^ soy agar with 5% sheep blood (TSBA) (Becton Dickinson GmbH, Heidelberg, Germany). Difco^TM^ LB broth, Lennox (LB-L) (Becton, Dickinson and Company, Sparks, USA). Midazolam (Hameln Pharma Plus GmbH, Hameln, Germany). Fentanyl (Hameln Pharma Plus GmbH, Hameln, Germany). Domitor^TM^ medetomidine hydrochloride (Orion Corporation, Espoo, Finnland). Naloxone hydrochloride (Inresa Arzneimtittel GmbH, Freiburg, Germany). Flumazenil (Inresa Arzneimtittel GmbH, Freiburg, Germany). Antisedan^TM^ atipamezole hydrochloride (Orion Corporation, Espoo, Finnland). Sodium chloride (VWR International Ltd., Leuven, Belgium). Chlorin e6 gel (using hydroxypropyl methylcellulose as gel-base) (by courtesy of APOCARE Pharma GmbH, Bielefeld, Germany). Methylene blue solution (Sigma-Aldrich, Steinheim, Germany). Phosphate-buffered saline (PBS) (Sigma-Aldrich, Steinheim, Germany).

### Bacterial strain and culture conditions

The MDR *P. aeruginosa* strain PA54, isolated from human infection, was collected in 2009 at the Institute of Medical Microbiology and Hygiene, Saarland University Medical Center, Homburg, Germany. The resistance profile of PA54 was determined using the automated VITEK 2 system (bioMérieux GmbH, Nürtingen, Germany). PA54 was routinely cultivated overnight on TSBA at 37 °C.

### Murine corneal scratch model

#### Mice

In preliminary experiments, we found that in mice younger than 6 months, *P. aeruginosa* keratitis showed a trend of spontaneous resolution during one week. Therefore, we used eight-to-eleven month old female C57BL/6N mice, which were purchased from Janvier (Le Genest Saint Isle, France) and kept under specific pathogen-free conditions according to the regulations of German veterinary law. All experiments were conducted in accordance with the German legislation on protection of animals and the NIH Guide for the Care and Use of Laboratory Animals (Institute of Laboratory Animal Resources, National Research Council, Washington, USA), and were approved by the local State Review Boards (Landesamt für Soziales, Gesundheit und Verbraucherschutz) of Saarland following the national guidelines for the ethical and humane treatment of animals.

#### Inoculum preparation

PA54 was grown overnight in LB-L at 37 °C, diluted in fresh medium in a ratio of 1:100, and incubated at 37 °C and 150 rpm shaking for 3 hours. Bacteria from the exponential growth phase were harvested by centrifugation, washed twice in PBS, resuspended in fresh LB-L to an optical density at 600 nm (OD_600_) of 10. The number of viable cells was determined by plating out serial dilutions on TSBA. CFU were counted after incubation overnight at 37 °C.

#### Infection

The time of infection of mice was deemed as hour 0 on day 0 of the experiment. Mice were narcotized by intraperitoneal injection of 10 μl/g anesthetic consisting of 5 μg/ml Fentanyl, 0.5 mg/ml midazolam and 0.05 mg/ml medetomidine hydrochloride in 0.9% sodium chloride solution. The natural tear flow was suppressed by dropping 10 μl of a 0.6% acetyl-cysteine solution onto the cornea. Subsequently, mice were placed under a stereo-microscope, and three linear scratches (each ~2 mm) were made on the ocular surface of the left eye of each mouse by a 27 gauge syringe needle to expose the subepithelial stroma. 5 μl of the PA54 suspension, corresponding to 1 × 10^6^ CFU, were pipetted onto the mechanically harmed cornea when mice were lateral recumbent. The bacterial solution was left on the eye for 20 minutes, and mice were subsequently awaked by injecting 10 μl/g antidote consisting of 0.12 mg/ml naloxone hydrochloride, 0.05 mg/ml flumazenil and 0.25 mg/ml atipamezole hydrochloride in 0.9% sodium chloride solution.

#### Grading of corneal infections

The corneal manifestation of the PA54 induced keratitis in the infected mouse eyes were examined daily under a stereomicroscope, and recorded as a clinical score using the following scale: Score 0, entire cornea clear; score 1, <50% cornea is semi-transparent (iris color behind the lesion can be identified); score 2, >50% cornea is semi-transparent; score 3, <50% cornea is opaque (iris color behind the lesion cannot be identified); score 4, >50% cornea is opaque; score 5, corneal spontaneous perforation or phthisis bulbi. Corneal photos were taken under the stereomicroscope when mice were in anesthesia.

#### Bacterial load determinations

Enucleated mouse eyes were homogenized with a POLYTRON PT 1200 E Manual Disperser (Kinematica AG, Lucerne, Switzerland) in 1 ml LB-L, and serial dilutions of this homogenate plated on TSBA. After 24 h of incubation at 37 °C, CFU were counted.

### PDI studies

**Figure 1:**
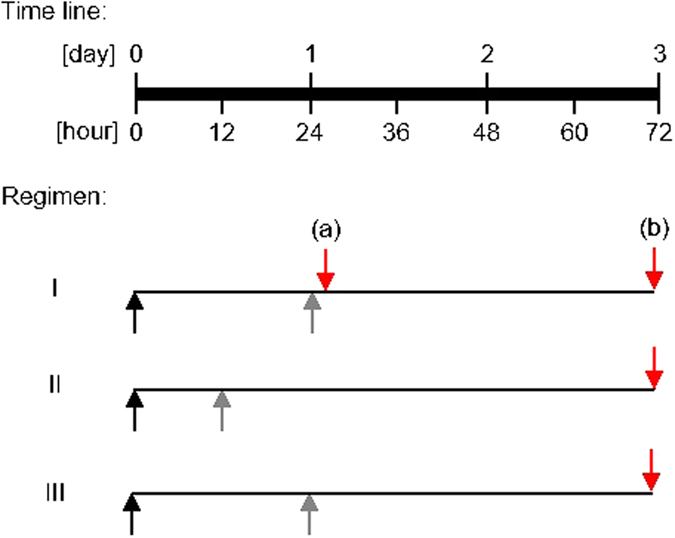
Schematic representation of key events applied in regimens I to III. Black arrows, tissue injury and infection; gray arrows, epithelium removal and PDI; red arrows, euthanasia, enucleation and CFU determinations. In regimen I, mice were either euthanized at 1 h post PDI (a) or 48 h post PDI (b).

**Figure 2:**
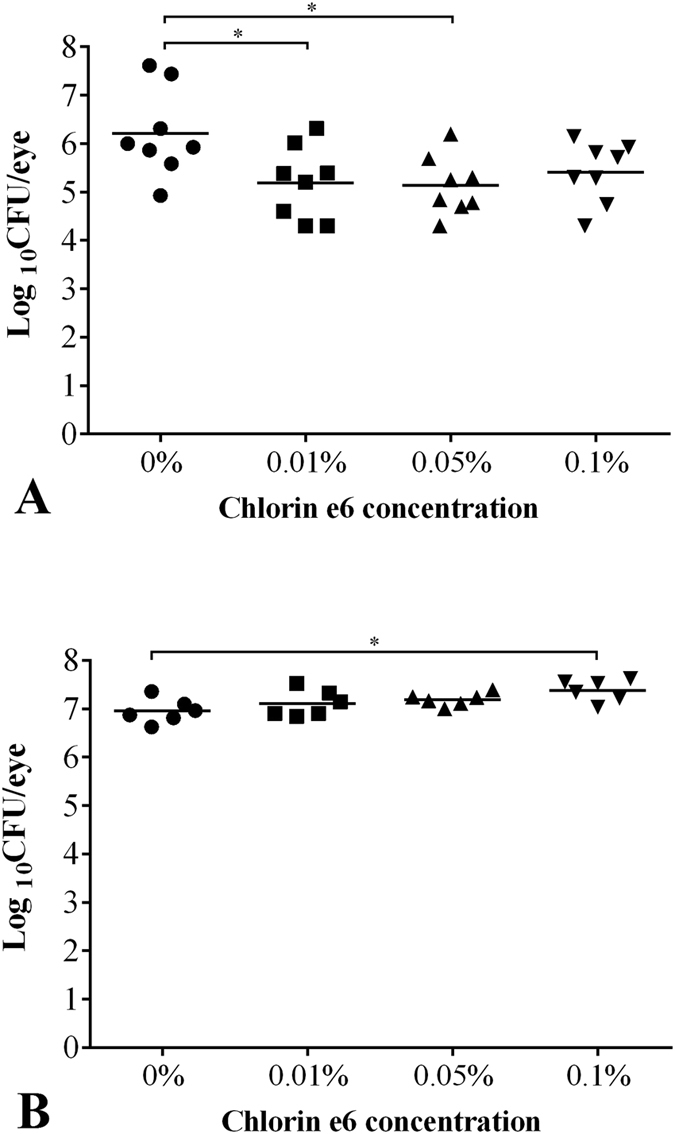
(**A**) Log_10_CFU 1 h after regimen I (chlorin e6 mediated PDI at 24 h post infection), (**B**) Log_10_CFU on day 3, of each mouse is displayed as a single dot in the scatter plot, grouped by the concentrations of chlorin e6 (Ce6) used in PDI at hour 24. Mean of Log_10_CFU of each group is shown as a bar. (**A**) Log_10_CFU in 0.01% Ce6 group (*P = 0.028) and 0.05% Ce6 group (*P = 0.015) are significantly lower than the control group treated by 0% Ce6. (**B**) Log_10_CFU in 0.1% Ce6 group is significantly higher than the control group treated by 0% Ce6 (*P = 0.011).

**Figure 3:**
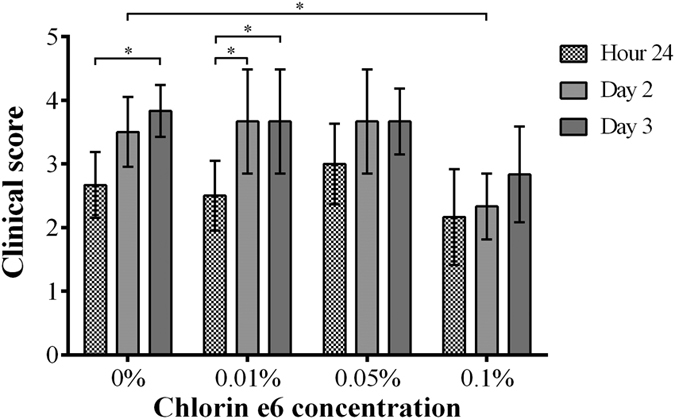
Clinical scores of mice treated by regimen I (chlorin e6 mediated PDI at 24 h post infection), recorded from hour 24 to day 3, grouped by the concentrations of chlorin e6 (Ce6) used in PDI. Means with standard deviations are shown. On day 2, clinical scores of 0.1% Ce6 group were significantly lower than the control group treated by 0% Ce6 (*P = 0.015). Clinical scores of 0% Ce6 group increased significantly from hour 24 to day 3 (*P = 0.009). Clinical scores of 0.01% Ce6 group increased significantly from hour 24 to day 2 (*P = 0.026), and from hour 24 to day 3 (*P = 0.026).

**Figure 4:**
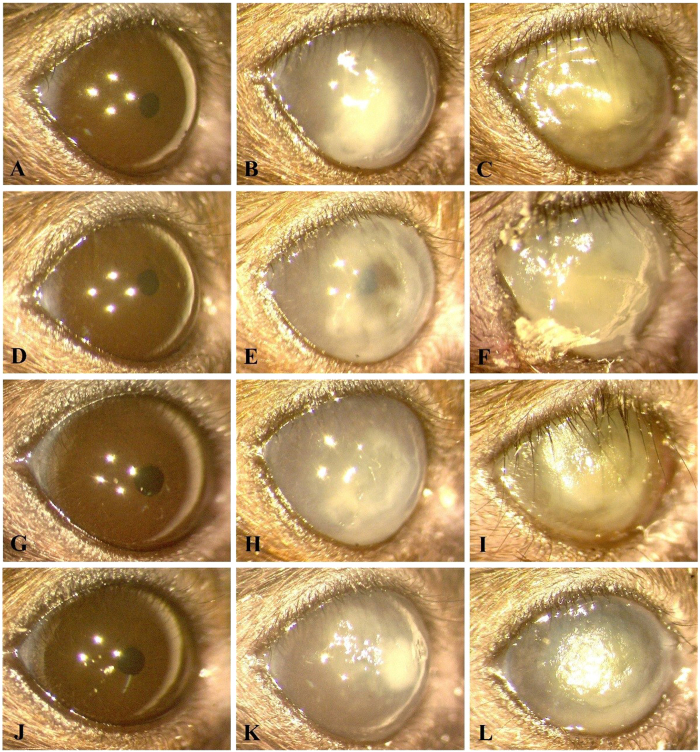
Representative corneal manifestations of mice treated by regimen I (chlorin e6 mediated PDI at 24 h post infection), in which PDI was mediated by (**A**,**B**,**C**) 0%, (**D**,**E**,**F**) 0.01%, (**G**,**H**,**I**) 0.05% and (**J**,**K**,**L**) 0.1% chlorin e6 (Ce6). Photos were taken (**A**,**D**,**G**,**J**) right before infection of *P. aeruginosa* (hour 0), (**B**,**E**,**H**,**K**) before PDI (hour 24), and (**C**,**F**,**I**,**L**) on day 3. (**E**) A characteristic infiltration ring of *P. aeruginosa* keratitis (clinical score 3). The corneal suppurative infiltration developed rapidly in all mice, but on day 3, it seemed to be milder in (**L**) the mouse treated by 0.1% Ce6 mediated PDI (clinical score 3) than the others (clinical score 4).

**Figure 5:**
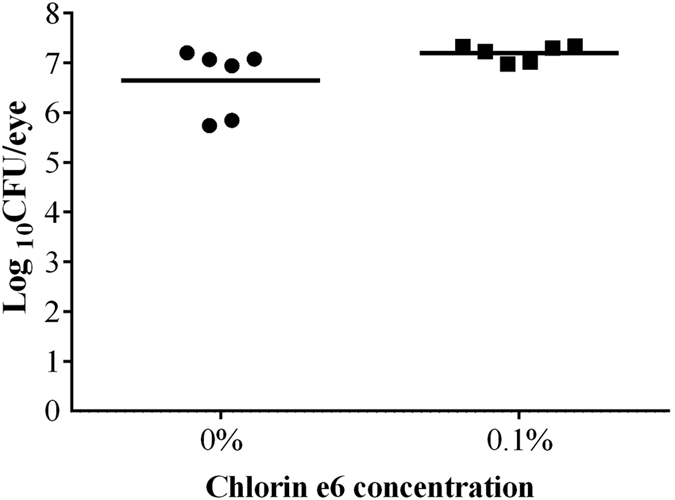
Log_10_CFU on day 3 of each mouse treated by regimen II (chlorin e6 mediated PDI at hour 12 post infection) is displayed as a single dot in the scatter plot. Mean of Log_10_CFU of each group is shown as a bar. 0.1% chlorin e6 (Ce6) was used in PDI at hour 12, while 0% Ce6 was used in control group. There was no significant difference between the two groups.

**Figure 6:**
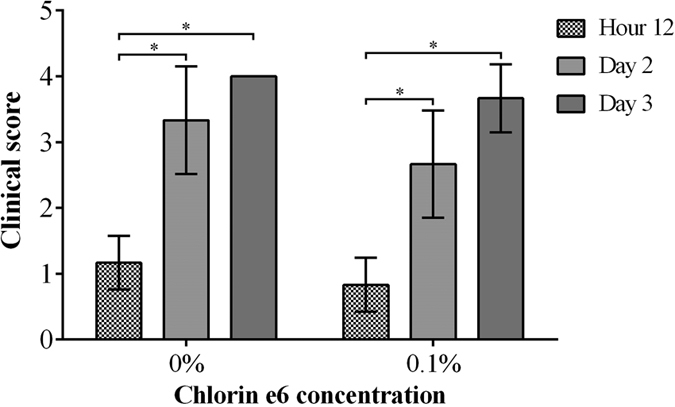
Clinical scores of mice treated by regimen II (chlorin e6 mediated PDI at hour 12 post infection), recorded from hour 12 to day 3. Means with standard deviations are shown. 0.1% chlorin e6 (Ce6) was used in PDI at hour 12, while 0% Ce6 was used in control group. There was no significant difference between the two groups. Clinical scores of 0% Ce6 group increased significantly from hour 12 to day 2 (*P = 0.002), and from hour 12 to day 3 (*P = 0.002). Similarly, clinical scores of 0.1% Ce6 group increased significantly from hour 12 to day 2 (*P = 0.002), and from hour 12 to day 3 (*P = 0.002).

**Figure 7:**
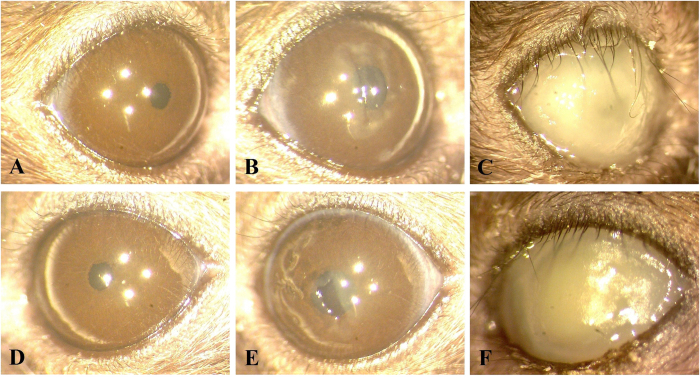
Representative corneal manifestations of mice treated by regimen II (chlorin e6 mediated PDI at hour 12 post infection), in which PDI was mediated by (**A**,**B**,**C**) 0%, (**D**,**E**,**F**) 0.1% chlorin e6 (Ce6). Photos were taken (**A**,**D**) right before infection of *P. aeruginosa* (hour 0), (**B**,**E**) before PDI (hour 12), and (**C**,**F**) on day 3. (**B**,**E**) At hour 12, corneal infiltrations were mild (clinical score 1), and the epithelial scratches made for infection of *P. aeruginosa* were still clearly seen. (**C**,**F**) The corneal infiltration was more severe in both mice on day 3 (clinical score 4).

**Figure 8:**
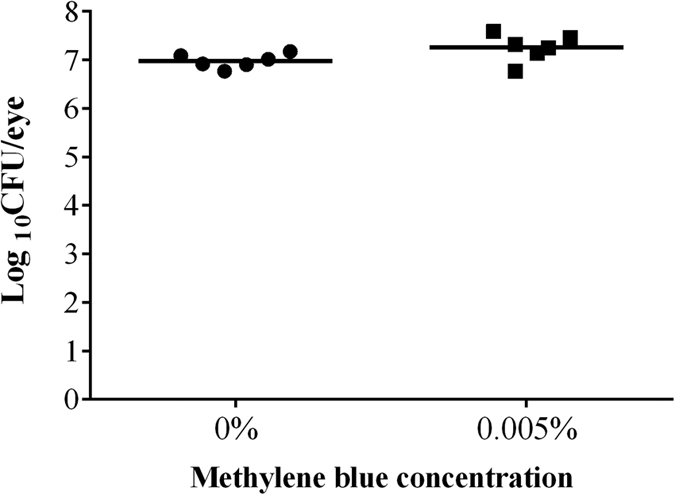
Log_10_CFU on day 3 of each mouse treated by regimen III (methylene blue mediated PDI at 24 h post infection) is displayed as a single dot in the scatter plot. Mean of Log_10_CFU of each group is shown as a bar. 0.005% methylene blue (MB) was used in PDI at hour 24, while 0% MB was used in control group. There was no significant difference between the two groups.

**Figure 9:**
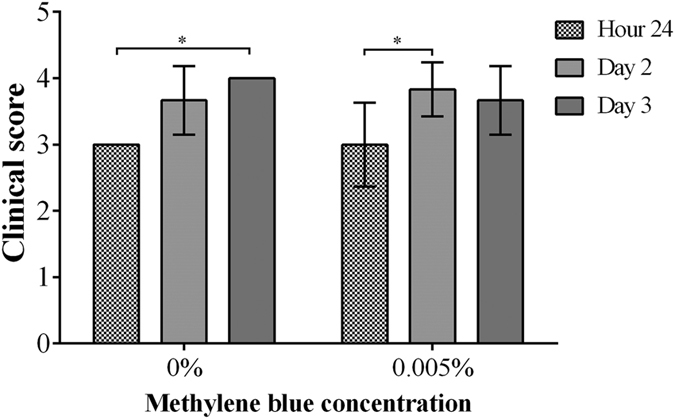
Clinical scores of mice treated by regimen III (methylene blue mediated PDI at 24 h post infection), recorded from hour 24 to day 3. Means with standard deviations are shown. 0.005% methylene blue (MB) was used in PDI at hour 24, while 0% MB was used in control group. There was no significant difference between the two groups. Clinical scores of 0% MB group increased significantly from hour 24 to day 3 (*P = 0.002). Clinical scores of 0.005% MB group increased significantly from hour 24 to day 2 (*P = 0.041).

**Figure 10:**
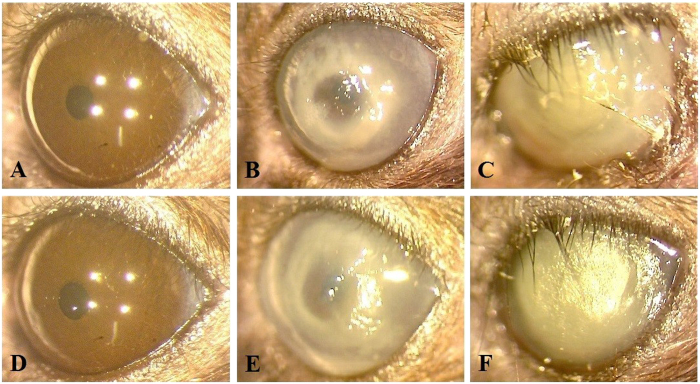
Representative corneal manifestations of mice treated by regimen III (methylene blue mediated PDI at 24 h post infection), in which PDI was mediated by (**A**,**B**,**C**) 0%, (**D**,**E**,**F**) 0.005% methylene blue (MB). Photos were taken (**A**,**D**) right before infection of *P. aeruginosa* (hour 0), (**B**,**E**) before PDI (hour 24), and (**C**,**F**) on day 3. (**B**,**E**) At hour 24, characteristic infiltration rings of *P. aeruginosa* keratitis were clearly seen (both clinical score 3). (**C**,**F**) The corneal infiltration was more severe in both mice on day 3 (clinical score 4).

All steps involving Ce6 were carried out in a dark room under weak yellow light exposure (λ = 596 nm) to avoid activation of the photosensitizer. Three regimens of PDI were tested (Fig. 1). In all regimens, corneal epithelia of narcotized mice were removed by a hockey epithelium removal knife prior to PDI. The irradiation setting to activate Ce6 was identical in all regimens, with 6 minutes irradiation of red light (λ = 670 nm), equivalent to an energy dose of 24 J/cm^2^.

#### Regimen I

At 24 h post infection, corneal epithelia were removed from infected eyes, and about 50 μl of a 0% (hydroxypropyl methylcellulose gel-base only), 0.01%, 0.05%, and 0.1% Ce6 gel dabbed onto the infected mouse cornea, respectively. Mice were then left in darkness for 30 minutes to allow the penetration of Ce6 into the corneal tissues. Afterwards, gel residues were carefully removed with cotton swabs, and mouse corneas irradiated as indicated above. Mice were euthanized either 1 h post PDI or 48 h post PDI, the infected eyes enucleated and subsequently homogenized for CFU determinations.

#### Regimen II

Corneal epithelia were removed from infected eyes at 12 h post infection, and about 50 μl of a 0% and 0.1% Ce6 gel dabbed onto the infected mouse cornea, respectively. The PDI treatment was carried out as described before. Mice were euthanized 60 h post PDI and handled as described above.

#### Regimen III

Corneal epithelia were removed from infected eyes at 24 h post infection, and about 20 μl of a 0.005% MB solution and PBS dropped onto the infected mouse cornea, respectively. The PDI treatment was carried out as described before. Mice were euthanized 48 h post PDI, and handled as described above.

### Statistical analysis

Statistical analysis was performed with IBM SPSS Statistics 22.0. Since bacterial growth data is a typical variable that follows the *log*-normal distribution^62^, after *log* transformation of the CFU values, independent samples t test following analysis of variance was used to compare Log_10_CFU. Mann-Whitney U test following Kruskal-Wallis test was used to compare clinical scores. *P* < 0.05 was considered statistically significant.

## Additional Information

**How to cite this article:** Wu, M.-F. *et al*. Chlorin e6 mediated photodynamic inactivation for multidrug resistant *Pseudomonas aeruginosa* keratitis in mice *in vivo. Sci. Rep.* **7**, 44537; doi: 10.1038/srep44537 (2017).

**Publisher's note:** Springer Nature remains neutral with regard to jurisdictional claims in published maps and institutional affiliations.

## References

[b1] Shah A., Sachdev A., Coggon D. & Hossain P. Geographic variations in microbial keratitis: an analysis of the peer-reviewed literature. Br J Ophthalmol 95, 762–767, doi: 10.1136/bjo.2009.169607 (2011). 21478201 PMC3403809

[b2] Hazlett L. D. Corneal response to Pseudomonas aeruginosa infection. Prog Retin Eye Res 23, 1–30, doi: 10.1016/j.preteyeres.2003.10.002 (2004). 14766315

[b3] Levy S. B. & Marshall B. Antibacterial resistance worldwide: causes, challenges and responses. Nat Med 10, S122–129, doi: 10.1038/nm1145 (2004). 15577930

[b4] Lister P. D., Wolter D. J. & Hanson N. D. Antibacterial-resistant Pseudomonas aeruginosa: clinical impact and complex regulation of chromosomally encoded resistance mechanisms. Clin Microbiol Rev 22, 582–610, doi: 10.1128/CMR.00040-09 (2009). 19822890 PMC2772362

[b5] McDonald E. M., Ram F. S., Patel D. V. & McGhee C. N. Topical antibiotics for the management of bacterial keratitis: an evidence-based review of high quality randomised controlled trials. Br J Ophthalmol 98, 1470–1477, doi: 10.1136/bjophthalmol-2013-304660 (2014). 24729078

[b6] Willcox M. D. Review of resistance of ocular isolates of Pseudomonas aeruginosa and staphylococci from keratitis to ciprofloxacin, gentamicin and cephalosporins. Clinical & experimental optometry 94, 161–168, doi: 10.1111/j.1444-0938.2010.00536.x (2011). 21083760

[b7] Vazirani J., Wurity S. & Ali M. H. Multidrug-Resistant Pseudomonas aeruginosa Keratitis: Risk Factors, Clinical Characteristics, and Outcomes. Ophthalmology 122, 2110–2114, doi: 10.1016/j.ophtha.2015.06.007 (2015). 26189185

[b8] Jain R., Murthy S. I. & Motukupally S. R. Clinical outcomes of corneal graft infections caused by multi-drug resistant Pseudomonas aeruginosa. Cornea 33, 22–26, doi: 10.1097/ICO.0000000000000011 (2014). 24240490

[b9] Wong R. L., Gangwani R. A., Yu L. W. & Lai J. S. New treatments for bacterial keratitis. Journal of ophthalmology 2012, 831502, doi: 10.1155/2012/831502 (2012). 22991650 PMC3444050

[b10] Szentmary N., Goebels S., Bischoff M. & Seitz B. [Photodynamic therapy for infectious keratitis]. Der Ophthalmologe: Zeitschrift der Deutschen Ophthalmologischen Gesellschaft 109, 165–170, doi: 10.1007/s00347-011-2511-x (2012). 22350551

[b11] Huang L. . Type I and Type II mechanisms of antimicrobial photodynamic therapy: an *in vitro* study on gram-negative and gram-positive bacteria. Lasers Surg Med 44, 490–499, doi: 10.1002/lsm.22045 (2012). 22760848 PMC3428129

[b12] Tavares A. . Antimicrobial photodynamic therapy: study of bacterial recovery viability and potential development of resistance after treatment. Mar Drugs 8, 91–105, doi: 10.3390/md8010091 (2010). 20161973 PMC2817925

[b13] Winckler K. D. Special section: focus on anti-microbial photodynamic therapy (PDT). J Photochem Photobiol B 86, 43–44, doi: 10.1016/j.jphotobiol.2006.09.005 (2007). 17067809

[b14] Pavan-Langston D. & Langston R. H. Recent advances in antiviral therapy. Int Ophthalmol Clin 15, 89–100 (1975). 818047 10.1097/00004397-197501540-00008

[b15] O’Day D. M. . Proflavine photodynamic viral inactivation in herpes simplex keratitis. Am J Ophthalmol 79, 941–948 (1975). 166561 10.1016/0002-9394(75)90675-3

[b16] Perlin M., Mao J. C., Otis E. R., Shipkowitz N. L. & Duff R. G. Photodynamic inactivation of influenza and herpes viruses by hematoporphyrin. Antiviral Res 7, 43–51 (1987). 3026246 10.1016/0166-3542(87)90038-6

[b17] Chen Z., Xuguang S., Zhiqun W. & Ran L. *In vitro* amoebacidal activity of photodynamic therapy on Acanthamoeba. Br J Ophthalmol 92, 1283–1286, doi: 10.1136/bjo.2007.134288 (2008). 18723746

[b18] Mito T. . Effect of photodynamic therapy with methylene blue on Acanthamoeba *in vitro*. Invest Ophthalmol Vis Sci 53, 6305–6313, doi: 10.1167/iovs.12-9828 (2012). 22918636

[b19] Kashiwabuchi R. T. . Assessment of fungal viability after long-wave ultraviolet light irradiation combined with riboflavin administration. Graefes Arch Clin Exp Ophthalmol 251, 521–527, doi: 10.1007/s00417-012-2209-z (2013). 23180236

[b20] Arboleda A. . Assessment of rose bengal versus riboflavin photodynamic therapy for inhibition of fungal keratitis isolates. Am J Ophthalmol 158, 64–70 e62, doi: 10.1016/j.ajo.2014.04.007 (2014). PMC4075940 24792103

[b21] Kashiwabuchi R. T. . Antimicrobial susceptibility of photodynamic therapy (UVA/riboflavin) against Staphylococcus aureus. Arq Bras Oftalmol 75, 423–426 (2012). 23715147 10.1590/s0004-27492012000600011

[b22] Halili F. . Rose bengal and riboflavin mediated photodynamic therapy to inhibit methicillin-resistant Staphylococcus aureus keratitis isolates. Am J Ophthalmol, doi: 10.1016/j.ajo.2016.03.014 (2016). 27016125

[b23] Kharkwal G. B., Sharma S. K., Huang Y. Y., Dai T. & Hamblin M. R. Photodynamic therapy for infections: clinical applications. Lasers Surg Med 43, 755–767, doi: 10.1002/lsm.21080 (2011). 22057503 PMC3449167

[b24] Raiskup F. & Spoerl E. Corneal crosslinking with riboflavin and ultraviolet A. I. Principles. Ocul Surf 11, 65–74, doi: 10.1016/j.jtos.2013.01.002 (2013). 23583042

[b25] Makdoumi K., Mortensen J. & Crafoord S. Infectious keratitis treated with corneal crosslinking. Cornea 29, 1353–1358, doi: 10.1097/ICO.0b013e3181d2de91 (2010). 21102196

[b26] Makdoumi K., Mortensen J., Sorkhabi O., Malmvall B. E. & Crafoord S. UVA-riboflavin photochemical therapy of bacterial keratitis: a pilot study. Graefes Arch Clin Exp Ophthalmol 250, 95–102, doi: 10.1007/s00417-011-1754-1 (2012). 21874347

[b27] Shetty R., Nagaraja H., Jayadev C., Shivanna Y. & Kugar T. Collagen crosslinking in the management of advanced non-resolving microbial keratitis. Br J Ophthalmol 98, 1033–1035, doi: 10.1136/bjophthalmol-2014-304944 (2014). 24711659

[b28] Chan E., Snibson G. R. & Sullivan L. Treatment of infectious keratitis with riboflavin and ultraviolet-A irradiation. J Cataract Refract Surg 40, 1919–1925, doi: 10.1016/j.jcrs.2014.09.001 (2014). 25262564

[b29] Kymionis G. D. . Herpetic keratitis with iritis after corneal crosslinking with riboflavin and ultraviolet A for keratoconus. J Cataract Refract Surg 33, 1982–1984, doi: 10.1016/j.jcrs.2007.06.036 (2007). 17964410

[b30] Pollhammer M. & Cursiefen C. Bacterial keratitis early after corneal crosslinking with riboflavin and ultraviolet-A. J Cataract Refract Surg 35, 588–589, doi: 10.1016/j.jcrs.2008.09.029 (2009). 19251154

[b31] Rama P., Di Matteo F., Matuska S., Paganoni G. & Spinelli A. Acanthamoeba keratitis with perforation after corneal crosslinking and bandage contact lens use. J Cataract Refr Surg 35, 788–791, doi: 10.1016/j.jcrs.2008.09.035 (2009). 19304108

[b32] Perez-Santonja J. J., Artola A., Javaloy J., Alio J. L. & Abad J. L. Microbial keratitis after corneal collagen crosslinking. J Cataract Refr Surg 35, 1138–1140, doi: 10.1016/j.jcrs.2009.01.036 (2009). 19465303

[b33] Sharma N., Maharana P., Singh G. & Titiyal J. S. Pseudomonas keratitis after collagen crosslinking for keratoconus: Case report and review of literature. J Cataract Refr Surg 36, 517–520, doi: 10.1016/j.jcrs.2009.08.041 (2010). 20202556

[b34] Ferrari G., Iuliano L., Vigano M. & Rama P. Impending corneal perforation after collagen cross-linking for herpetic keratitis. J Cataract Refr Surg 39, 638–641, doi: 10.1016/j.jcrs.2013.02.006 (2013). 23522585

[b35] Gautam, Jhanji V., Satpathy G., Khokhar S. & Agarwal T. Microsporidial keratitis after collagen cross-linking. Ocul Immunol Inflamm 21, 495–497, doi: 10.3109/09273948.2013.824105 (2013). 23978264

[b36] Rana M., Lau A., Aralikatti A. & Shah S. Severe microbial keratitis and associated perforation after corneal crosslinking for keratoconus. Cont Lens Anterior Eye 38, 134–137, doi: 10.1016/j.clae.2014.10.004 (2015). 25435381

[b37] Cagil N., Sarac O., Cakmak H. B., Can G. & Can E. Mechanical epithelial removal followed by corneal collagen crosslinking in progressive keratoconus: short-term complications. J Cataract Refract Surg 41, 1730–1737, doi: 10.1016/j.jcrs.2014.12.058 (2015). 26432132

[b38] Kodavoor S. K., Sarwate N. J. & Ramamurhy D. Microbial keratitis following accelerated corneal collagen cross-linking. Oman J Ophthalmol 8, 111–113, doi: 10.4103/0974-620X.159259 (2015). 26622139 PMC4640035

[b39] Shih M. H. & Huang F. C. Effects of photodynamic therapy on rapidly growing nontuberculous mycobacteria keratitis. Invest Ophthalmol Vis Sci 52, 223–229, doi: 10.1167/iovs.10-5593 (2011). 20811055

[b40] El-Laithy H. M., Nesseem D. I., El-Adly A. A. & Shoukry M. Moxifloxacin-Gelrite *in situ* ophthalmic gelling system against photodynamic therapy for treatment of bacterial corneal inflammation. Arch Pharm Res 34, 1663–1678, doi: 10.1007/s12272-011-1011-5 (2011). 22076767

[b41] Park J. H. . Antimicrobial effect of photodynamic therapy using a highly pure chlorin e6. Laser Med Sci 25, 705–710, doi: 10.1007/s10103-010-0781-1 (2010). 20414708

[b42] Tegos G. P. . Protease-stable polycationic photosensitizer conjugates between polyethyleneimine and chlorin(e6) for broad-spectrum antimicrobial photoinactivation. Antimicrob Agents Ch 50, 1402–1410, doi: 10.1128/Aac.50.4.1402-1410.2006 (2006). PMC1426948 16569858

[b43] Schastak S., Gitter B., Handzel R., Hermann R. & Wiedemann P. Improved photoinactivation of gram-negative and gram-positive methicillin-resistant bacterial strains using a new near-infrared absorbing meso-tetrahydroporphyrin: a comparative study with a chlorine e6 photosensitizer photolon. Methods and findings in experimental and clinical pharmacology 30, 129–133, doi: 10.1358/mf.2008.30.2.1165448 (2008). 18560628

[b44] Winkler K. . *In Vitro* Effectiveness Of Photodynamic Therapy Against Multi-resistant Pathogens. Investigative Ophthalmology & Visual Science 53, 6206–6206 (2012).

[b45] Ochsner M. Photophysical and photobiological processes in the photodynamic therapy of tumours. J Photochem Photobiol B 39, 1–18 (1997). 9210318 10.1016/s1011-1344(96)07428-3

[b46] Bergendi L., Benes L., Durackova Z. & Ferencik M. Chemistry, physiology and pathology of free radicals. Life Sci 65, 1865–1874 (1999). 10576429 10.1016/s0024-3205(99)00439-7

[b47] Griffiths H. R., Mistry P., Herbert K. E. & Lunec J. Molecular and cellular effects of ultraviolet light-induced genotoxicity. Crit Rev Cl Lab Sci 35, 189–237, doi: 10.1080/10408369891234192 (1998). 9663376

[b48] Winkler K. . Photodynamic inactivation of multidrug-resistant Staphylococcus aureus by chlorin e6 and red light (lambda = 670nm). J Photochem Photobiol B 162, 340–347, doi: 10.1016/j.jphotobiol.2016.07.007 (2016). 27419618

[b49] Tanaka M. . Optimal Photosensitizers for Photodynamic Therapy of Infections Should Kill Bacteria but Spare Neutrophils. Photochem Photobiol 88, 227–232, doi: 10.1111/j.1751-1097.2011.01005.x (2012). 21950417 PMC3253242

[b50] Simon C., Wolf G, Walther M, . Photodynamic inactivation of pathogens causing infectious keratitis. SPIE; 8931:89310T (2014).

[b51] Seiler T. G. . Two-Photon Fluorescence Microscopy for Determination of the Riboflavin Concentration in the Anterior Corneal Stroma When Using the Dresden Protocol. Investigative Ophthalmology & Visual Science 56, 6740–6746, doi: 10.1167/iovs.15-17656 (2015). 26567785

[b52] Gad F., Zahra T., Hasan T. & Hamblin M. R. Effects of growth phase and extracellular slime on photodynamic inactivation of gram-positive pathogenic bacteria. Antimicrob Agents Chemother 48, 2173–2178, doi: 10.1128/AAC.48.6.2173-2178.2004 (2004). 15155218 PMC415578

[b53] Dai T., Huang Y. Y. & Hamblin M. R. Photodynamic therapy for localized infections–state of the art. Photodiagnosis Photodyn Ther 6, 170–188, doi: 10.1016/j.pdpdt.2009.10.008 (2009). 19932449 PMC2811240

[b54] Hancock R. E. Resistance mechanisms in Pseudomonas aeruginosa and other nonfermentative gram-negative bacteria. Clin Infect Dis 27 Suppl 1, S93–99 (1998). 9710677 10.1086/514909

[b55] Hamblin M. R. . Polycationic photosensitizer conjugates: effects of chain length and Gram classification on the photodynamic inactivation of bacteria. J Antimicrob Chemother 49, 941–951 (2002). 12039886 10.1093/jac/dkf053

[b56] Long Y. . Novel polymeric nanoparticles targeting the lipopolysaccharides of Pseudomonas aeruginosa. Int J Pharm 502, 232–241, doi: 10.1016/j.ijpharm.2016.02.021 (2016). 26899978

[b57] Rice D. R., Gan H. Y. & Smith B. D. Bacterial imaging and photodynamic inactivation using zinc(II)-dipicolylamine BODIPY conjugates. Photoch Photobio Sci 14, 1271–1281, doi: 10.1039/c5pp00100e (2015). PMC4490101 26063101

[b58] Li K. . Selective Photodynamic Inactivation of Bacterial Cells over Mammalian Cells by New Triarylmethanes. Langmuir 30, 14573–14580, doi: 10.1021/la5028724 (2014). 25419964

[b59] Thibodeaux B. A. . Quantitative comparison of fluoroquinolone therapies of experimental gram-negative bacterial keratitis. Curr Eye Res 28, 337–342 (2004). 15287370 10.1076/ceyr.28.5.337.28676

[b60] Fukuda K. . Pseudomonas aeruginosa keratitis in mice: effects of topical bacteriophage KPP12 administration. PLoS One 7, e47742, doi: 10.1371/journal.pone.0047742 (2012). 23082205 PMC3474789

[b61] Kolar S. S. . Esculentin-1a(1-21)NH2: a frog skin-derived peptide for microbial keratitis. Cell Mol Life Sci 72, 617–627, doi: 10.1007/s00018-014-1694-0 (2015). 25086859 PMC4392908

[b62] Limpert E., Stahel W. A. & Abbt M. Log-normal distributions across the sciences: Keys and clues. Bioscience 51, 341–352, doi: 10.1641/0006-3568(2001)051[0341:Lndats]2.0.Co;2 (2001).

